# Supplementary material for: Snail determines the therapeutic response to mTOR kinase inhibitors by transcriptional repression of 4E-BP1
Source: Nat Commun. 2017 Dec 20;8:2207. doi: 10.1038/s41467-017-02243-3 (PMC5738350; doi:10.1038/s41467-017-02243-3)
Supplement: Supplementary file 1 — Supplementary Information [file 41467_2017_2243_MOESM1_ESM.pdf]

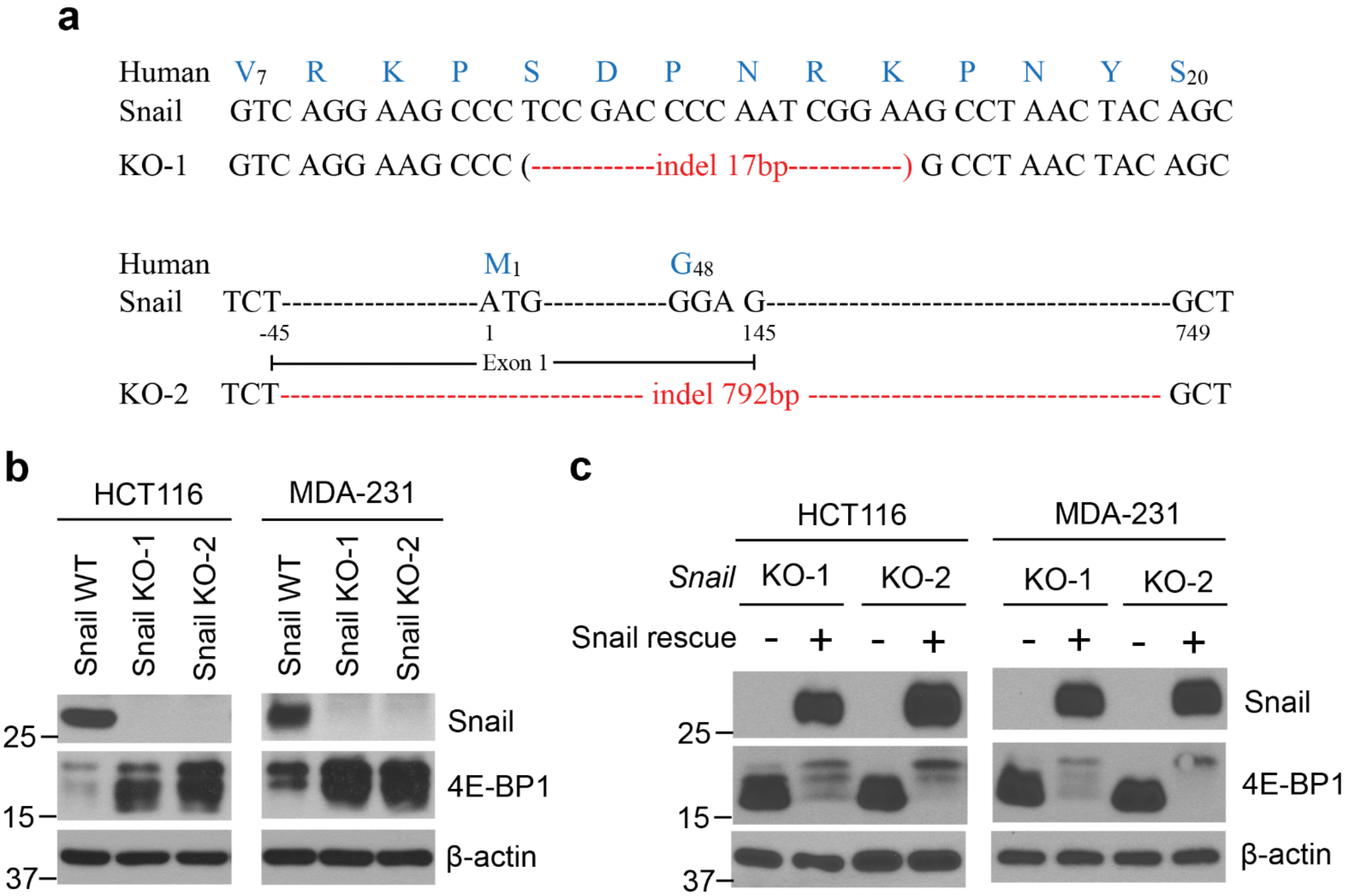

Supplementary Figure 1. Knockout of *Snail* profoundly induces 4E-BP1 expression. (**a**) Generation of human *Snail* knockout cell lines. Exon 1 of human *Snail* was targeted by the CRISPR-Cas9 nickase system. The sequences of internal deletion (indel) are shown in the two HCT116 or MDA-231 *Snail* knockout (KO) cell clones used in the study. Amino acid codes are indicated with blue characters. (**b**) Two *Snail* KO HCT116 or MDA-231 clones and their control wild type (WT) cells were analyzed by western blotting for the indicated proteins. (**c**) HCT116 or MDA-231 *Snail* KO cells were transiently infected with lentivirus using pLenti6.3-human Snail construct or vector control. After 48 h infection, cells were lysed and analyzed by western blotting for the indicated proteins.

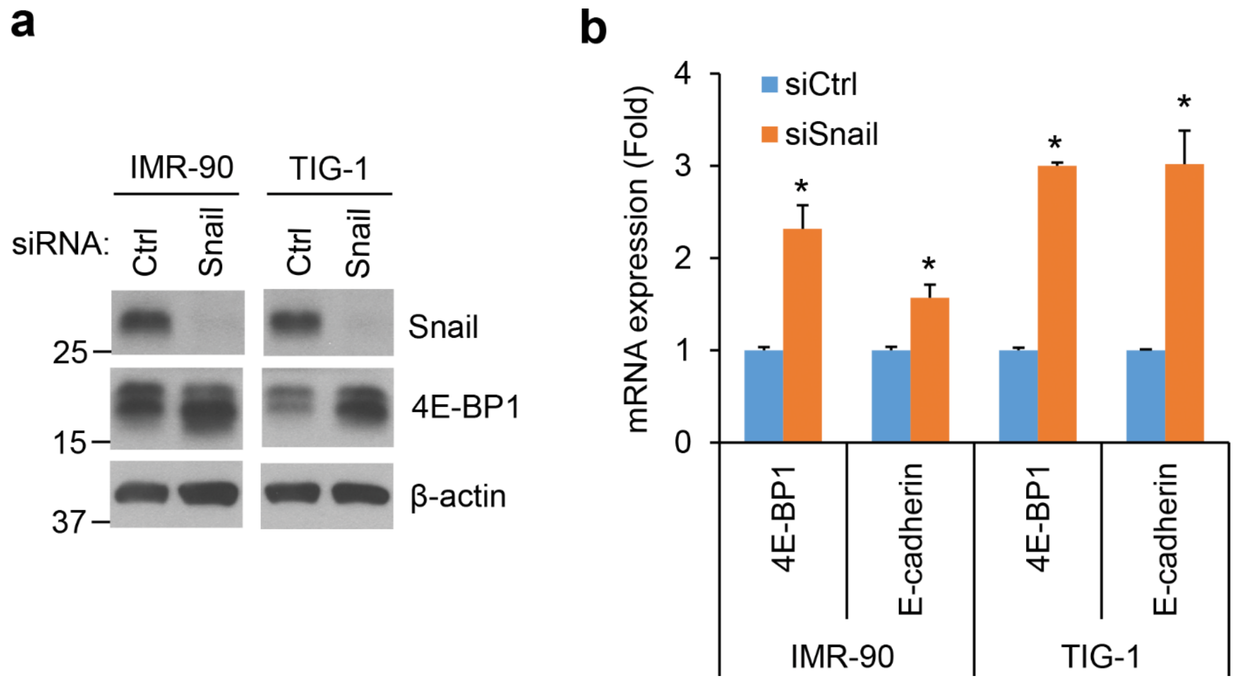

Supplementary Figure 2. Silencing Snail in normal human fibroblasts dramatically increases 4E-BP1 expression. IMR-90 and TIG1 normal human fetal lung fibroblasts were transfected with 20 nM of an siRNA pool targeting Snail (siSnail) or with a control siRNA (siCtrl) pool for 48 h, followed by western blot analysis for the indicated proteins (**a**), or by quantitative RT-PCR analysis for mRNA expression of 4E-BP1 and E-cadherin relative to the levels found in control siRNA cells (**b**). Data are presented as mean ± SEM (n=3 technical replicates per condition). $^{*}P < 0.001$ for siSnail vs. siCtrl using Student's *t*-test.

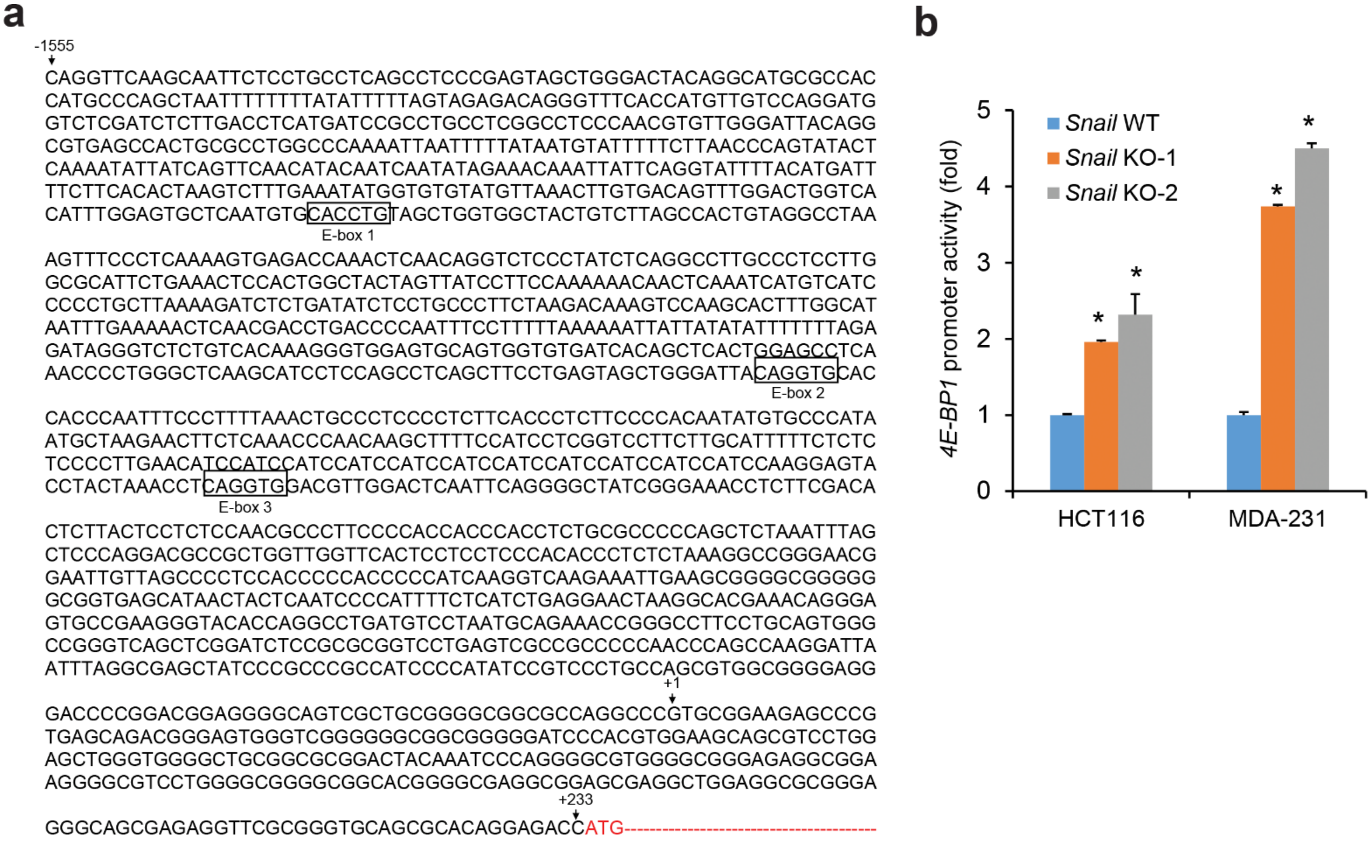

Supplementary Figure 3. Knockout of human *Snail* gene increases 4E-BP1 transcription activity. (a) Sequence (-1555 to +233) in the human 4E-BP1 promoter region. Position +1 indicates the location of the putative transcription start site (Ensembl genome browser, http://www.ensembl.org). The human 4E-BP1 promoter contains three putative Snail-binding E-boxes (CACCTG or CAGGTG) upstream of its transcription start site. Protein coding region is shown in red. (**b**) Two *Snail* KO HCT116 or MDA-231 clones (KO-1 and -2) and their *Snail* WT control cells were co-transfected with human 4E-BP1-Luc and an internal control *Renilla*-Luc. After 36 h transfection, cells were harvested and assessed for dual-luciferase activity. The firefly luciferase activity was normalized to *Renilla* and presented as a fold increase over the Luc activity found in *Snail* WT cells. Data are presented as mean ± SEM (n=3 technical replicates per condition). *$P < 0.001$ for *Snail* KO vs. *Snail* WT using Student's *t*-test.

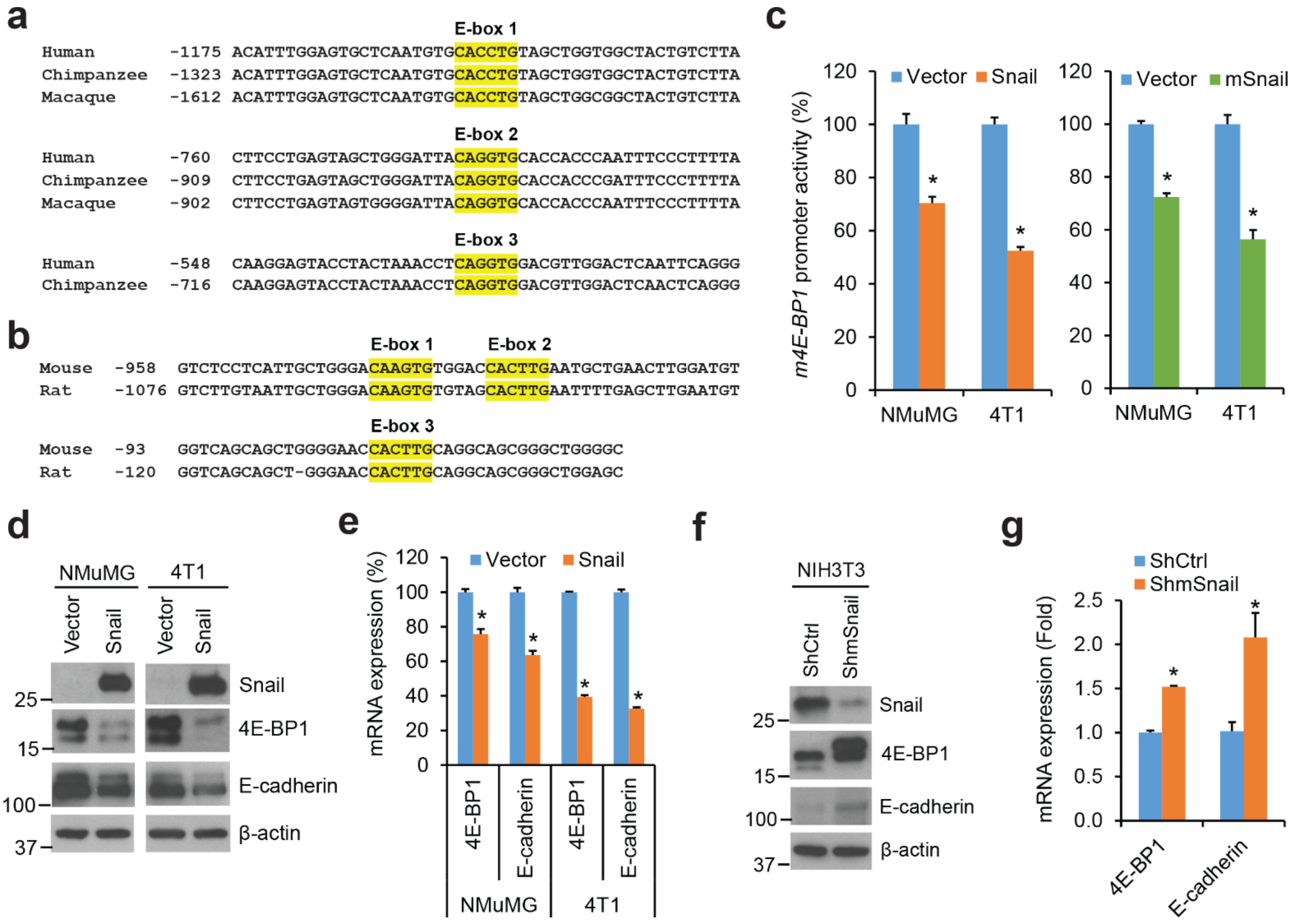

Supplementary Figure 4. Snail represses the mouse *4E-BP1* promoter activity and its expression. (**a, b**) Alignment of the conserved Snail-binding E-boxes (yellow) in *4E-BP1* promoters. The number at the start of the sequence indicates the distance from transcription start sites. (**c**) *Left*, NMuMG and 4T1 cells were transiently infected with lentivirus using pLenti6.3-human Snail or the empty vector construct for 36 h and then co-transfected with the mouse *4E-BP1* promoter-Luc and an internal control *Renilla*-Luc for an additional 36 h. *Right*, NMuMG and 4T1 cells were transfected with mouse Snail expression vector or the empty vector together with the mouse *4E-BP1* promoter-Luc and an internal control *Renilla*-Luc for 36 h. Dual-luciferase activities were assayed after transfection. The firefly luciferase activity was normalized to *Renilla* and presented as a percentage of the activity found in vector control cells. (**d, e**) NMuMG and 4T1 cells were transiently infected with lentivirus using pLenti6.3-human Snail or the empty vector construct for 48 h, followed by western blot analysis for the indicated proteins (**d**), or by quantitative RT-PCR analysis for mRNA expression of 4E-BP1 and E-cadherin relative to the levels found in vector control cells (**e**). (**f, g**) NIH3T3 cells with stable expression of mouse Snail shRNA (ShmSnail) or control shRNA (ShCtrl) were analyzed by western blotting for the indicated proteins (**f**), or by quantitative RT-PCR analysis for mRNA expression of 4E-BP1 and E-cadherin relative to the levels found in ShCtrl cells (**g**). All graphic data are presented as mean ± SEM (n=3 technical replicates per condition). $^{*}P < 0.001$ for Snail vs. vector or ShmSnail vs. ShCtrl using Student's *t*-test.

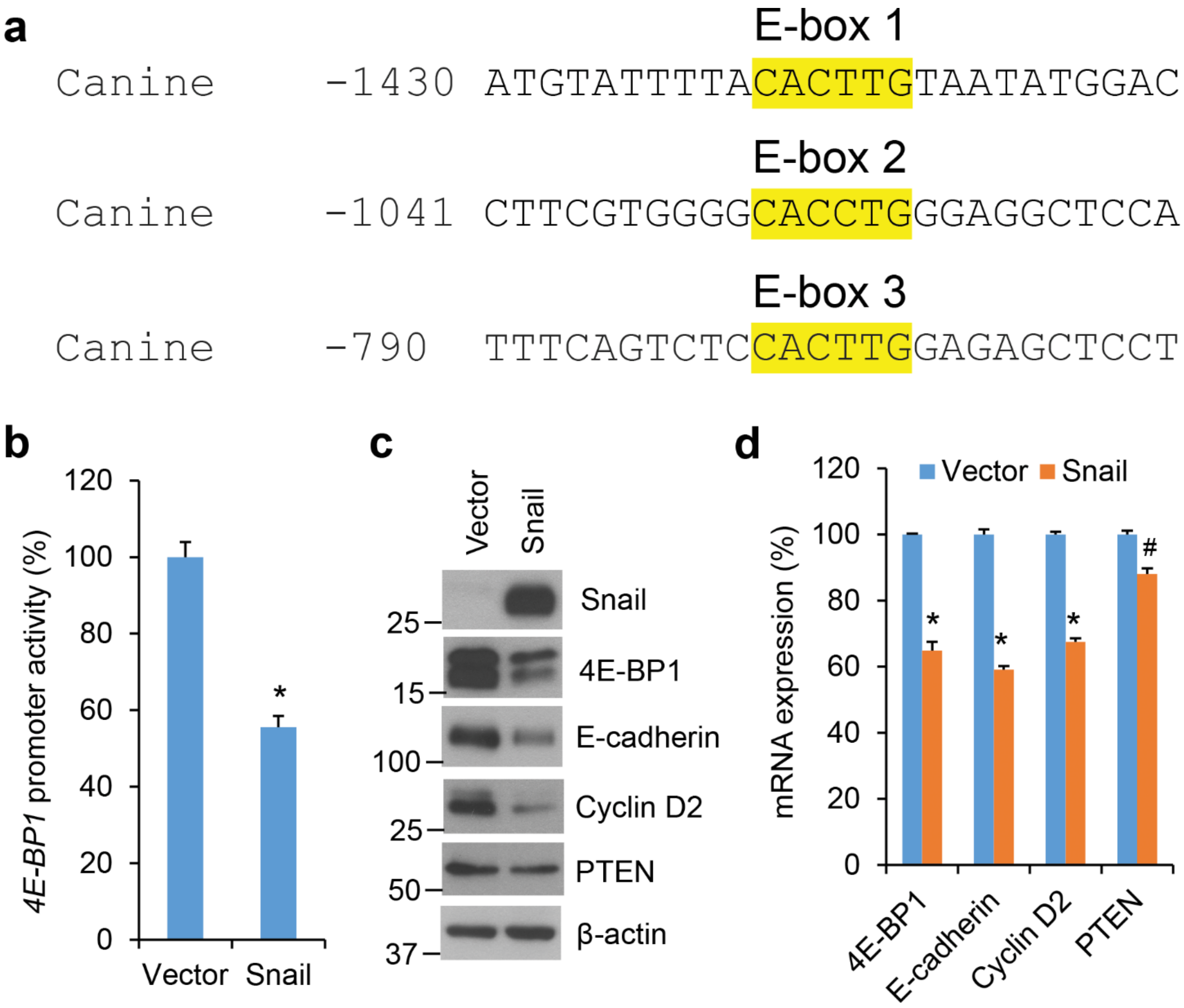

Supplementary Figure 5. Snail represses expression of 4E-BP1 and other Snail targets in MDCK cells. (**a**) The canine *4E-BP1* promoter contains three putative Snail-binding E-boxes (CACCTG or CACTTG). The number at the start of the sequence indicates the distance from transcription start sites. (**b**) MDCK cells were transiently infected with lentivirus using pLenti6.3-human Snail or with the empty vector construct for 36 h and then co-transfected with the human *4E-BP1* promoter-Luc and an internal control *Renilla*-Luc for an additional 24 h. Dual-luciferase activities were assayed after transfection. The firefly luciferase activity was normalized to *Renilla* and presented as a percentage of the activity found in vector control cells. (**c, d**) MDCK cells were transiently infected with lentivirus using pLenti6.3-human Snail or with the empty vector construct for 48, followed by western blot analysis for the indicated proteins (**c**), or by quantitative RT-PCR analysis for mRNA expression of the indicated genes relative to the levels found in vector control cells (**d**). All graphic data are presented as mean ± SEM (n=3 technical replicates per condition). $^{*}P < 0.001$; $^{\#}P < 0.02$ for Snail vs. vector using Student's *t*-test.

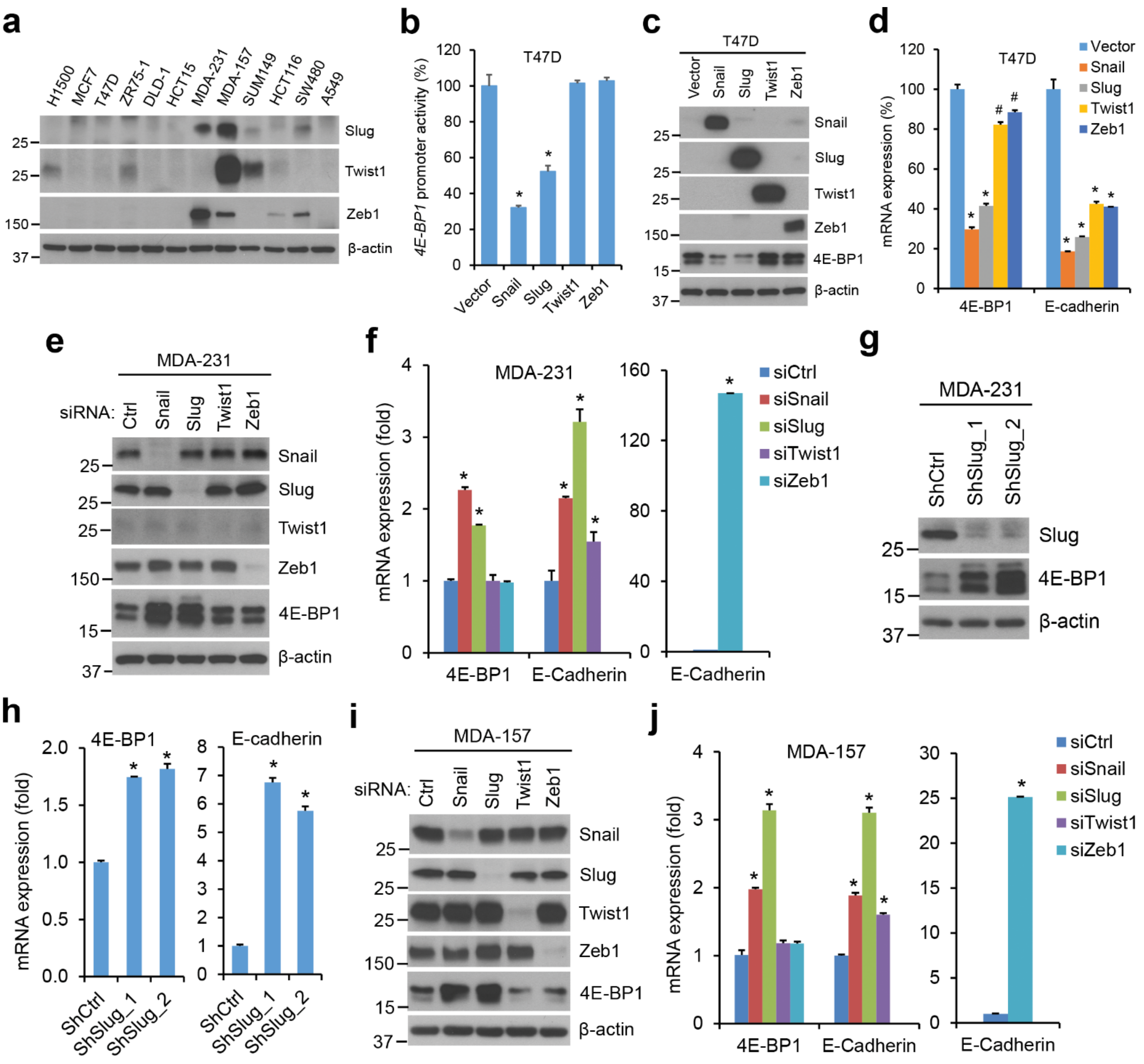

Supplementary Figure 6. Slug strongly represses 4E-BP1 promoter activity and its expression. (**a**) Cell lysates from the indicated cancer cell lines were analyzed by western blotting for the indicated proteins. (**b**) T47D cells were transiently infected with lentivirus using pLenti6.3-human Snail, Slug, Twist1, Zeb1 or with the empty vector construct for 36 h and then co-transfected with the human *4E-BP1* promoter-Luc and an internal control *Renilla*-Luc for an additional 24 h. Dual-luciferase activities were assayed after transfection. The firefly luciferase activity was normalized to *Renilla* and presented as a percentage of the activity found in vector control cells. (**c, d**) T47D cells were transiently infected with lentivirus using pLenti6.3-human Snail, Slug, Twist1, Zeb1 or with the empty vector plasmid construct for 48 h, followed by western blot analysis for the indicated proteins (**c**), or by quantitative RT-PCR analysis for mRNA expression of 4E-BP1 and E-cadherin relative to the levels found in vector control cells (**d**). (**e, f**) MDA-231 cells were transfected with 20 nM of an siRNA pool targeting human Snail, Slug, Twist1, Zeb1 or with the control siRNA pool. After a 48 h transfection, cells were assessed by western blot analysis for the indicated proteins (**e**), or by quantitative RT-PCR analysis for mRNA expression of 4E-BP1 and E-cadherin

relative to the levels found in control siRNA cells (**f**). (**g, h**) MDA-231 cells with stable expression of two different sets of Slug shRNAs (ShSlug_1 and ShSlug_2) or with control shRNA (ShCtrl) were assessed by western blot analysis for the indicated proteins (**g**), or by quantitative RT-PCR analysis for mRNA expression of 4E-BP1 and E-cadherin relative to the levels in ShCtrl cells (**h**). (**i, j**) MDA-157 cells were transfected with 20 nM of an siRNA pool targeting human Snail, Slug, Twist1, Zeb1 or with a control siRNA pool. After a 48 h transfection, cells were assessed by western blot analysis for the indicated proteins (**i**), or by quantitative RT-PCR analysis for mRNA expression of 4E-BP1 and E-cadherin relative to the levels found in control siRNA cells (**j**). All graphic data are presented as mean ± SEM (n=3 technical replicates per condition). $^{*}P < 0.001$; $^{\#}P < 0.02$ for the indicated genes vs. vector or ShSlug vs. ShCtrl using Student's *t*-test.

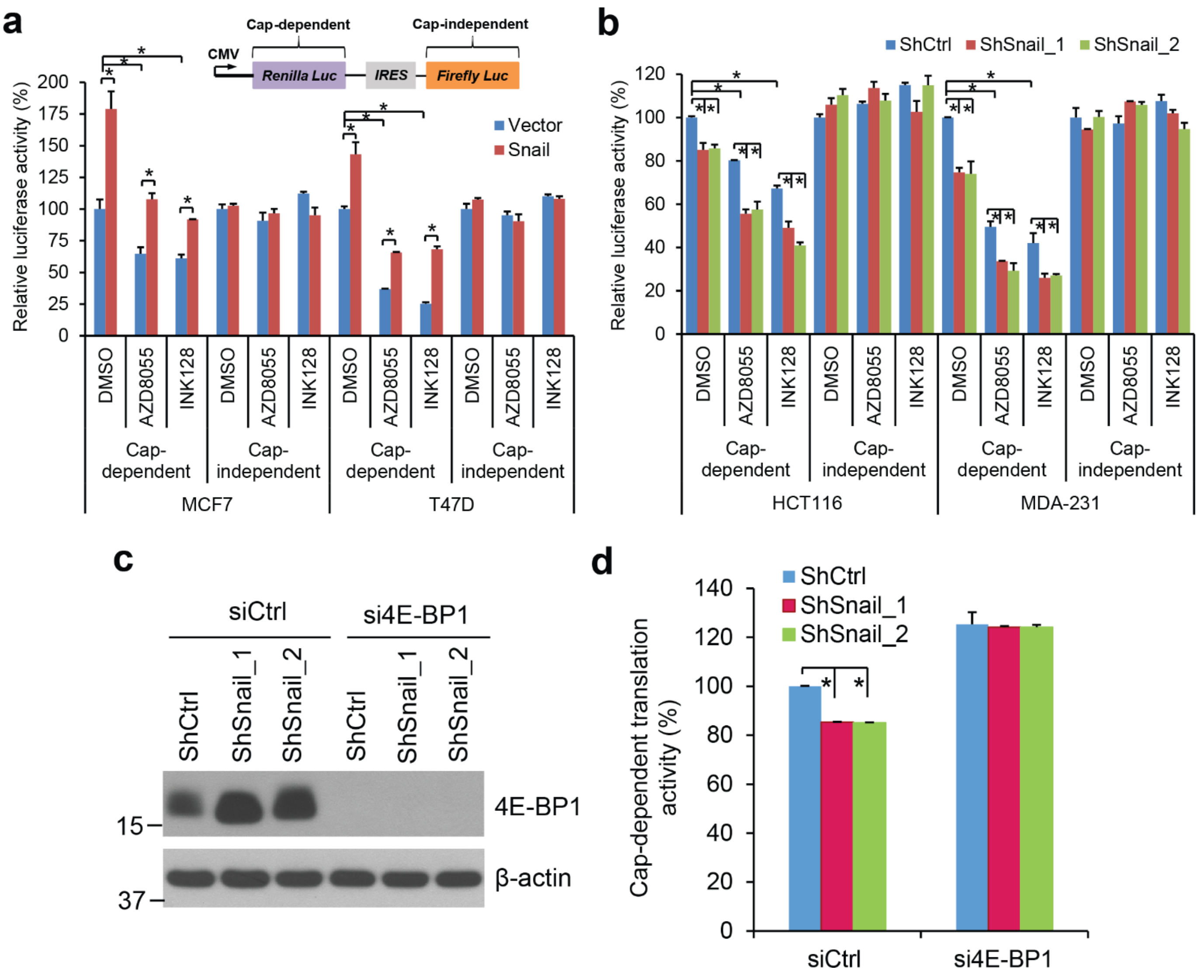

Supplementary Figure 7. Snail-mediated 4E-BP1 repression modulates cap-dependent but not cap-independent translation in response to mTORkis. (**a, b**) *Upper:* A diagram of the bicistronic reporter construct. The indicated cells with stable expression (**a**) or knockdown (**b**) of Snail or their respective controls, were transfected with a bicistronic luciferase reporter that detects cap-dependent translation of the *Renilla* luciferase gene as well as cap-independent poliovirus IRES-mediated translation of the firefly luciferase gene. After 24 h transfection, cells were treated with mTOR kinase inhibitors AZD8055 (100 nM), INK128 (100 nM) or DMSO as control for an additional 12 h. Each luciferase activity was measured using a dual-luciferase assay kit, and presented as a percentage of the activity found in the DMSO-treated vector control (**a**) or shRNA control (**b**) cells. (**c, d**) HCT116 cells with stable expression of Snail shRNA or with control shRNA were transfected with 20 nM control siRNA or 4E-BP1 siRNA for 48 h, followed by western blot analysis for the indicated proteins (**c**), or by the dual-luciferase assay (**d**). The ratio of *Renilla*/firefly luciferase activities was calculated and presented as a percentage of the cap-dependent translation activity found in the shRNA control cells. All graphic data are presented as mean ± SEM (n=3 technical replicates per condition). $^{*}P < 0.01$ using Student's *t*-test.

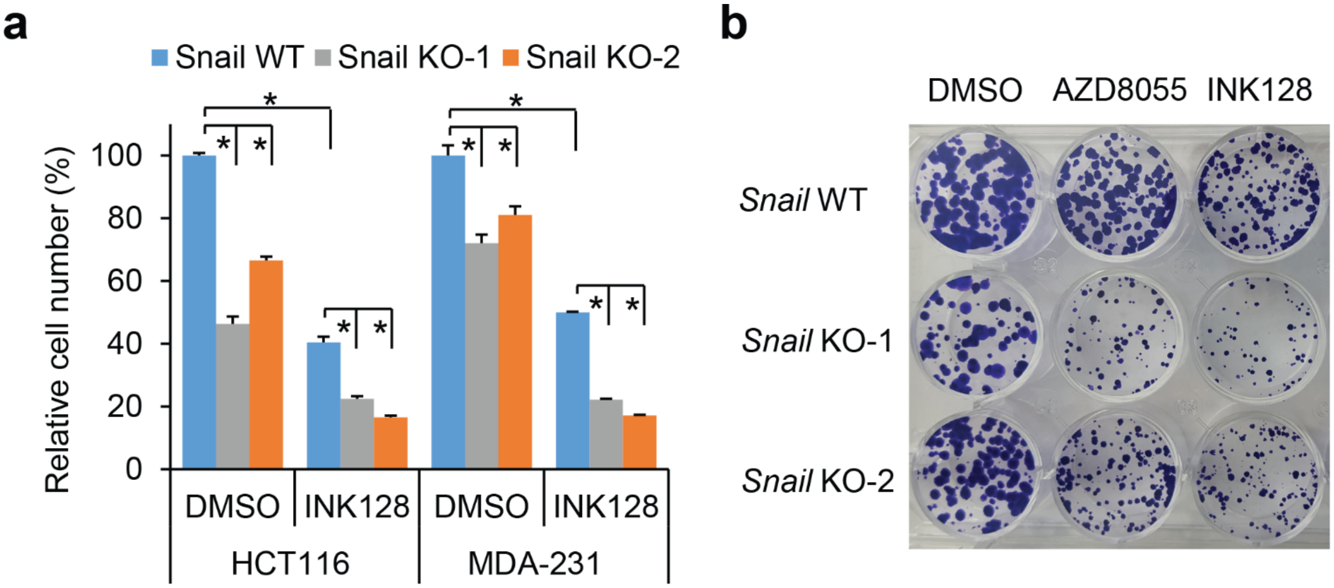

Supplementary Figure 8. Knockout of the *Snail* gene enhances inhibition of cell growth induced by mTOkis. (**a**) The growth of the indicated cells with *Snail* WT or KO was assessed after 72 h of treatment with 100 nM INK128 or with DMSO as control. The results are expressed as a percentage of cell number relative to values obtained in the DMSO-treated *Snail* WT cells. Data are presented as mean ± SEM (n=3 technical replicates per condition). $^{*}P < 0.001$ using Student's *t*-test. (**b**) Two *Snail* KO HCT116 clones and their *Snail* WT control cells (500 cells/well) were treated with 100 nM AZD8055, 100 nM INK128 or DMSO as control every 3 days. After 12 days of treatment, the formation of cell colonies was observed using crystal violet staining.

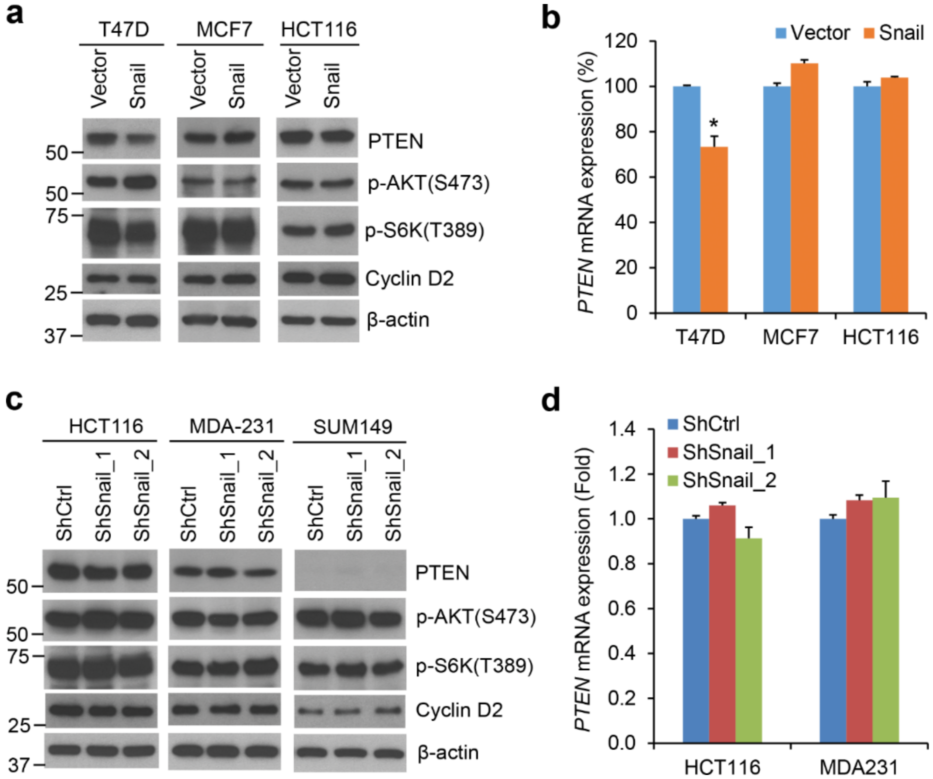

Supplementary Figure 9. Effect of Snail on the expression of PTEN and cyclin D2. (**a, b**) T47D, MCF7 and HCT116 cells with stable expression of Snail or with vector control were assessed by western blot analysis for the indicated proteins (**a**), or by quantitative RT-PCR analysis for mRNA expression of PTEN relative to the levels found in vector control cells (**b**). (**c, d**) HCT116, MDA-231 and SUM149 cells with stable expression of two different sets of Snail shRNAs (ShSnail_1 and ShSnail_2) or with control shRNA (ShCtrl) were assessed by western blot analysis for the indicated proteins (**c**), or by quantitative RT-PCR analysis for mRNA expression of PTEN relative to the levels found in ShCtrl cells (**d**). All graphic data are presented as mean ± SEM (n=3 technical replicates per condition). $^{*}P < 0.01$ for Snail vs. vector using Student's *t*-test.

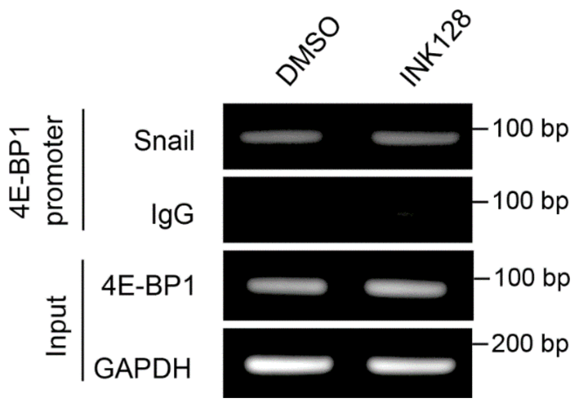

Supplementary Figure 10. Effect of INK128 on the level of Snail bound to the human 4E-BP1 promoter. HCT116 cells were treated with 100 nM INK128 for 12 h, followed by ChIP analysis using a specific antibody against Snail or with an irrelevant IgG and the set 3 primers described in the Figure 3d.

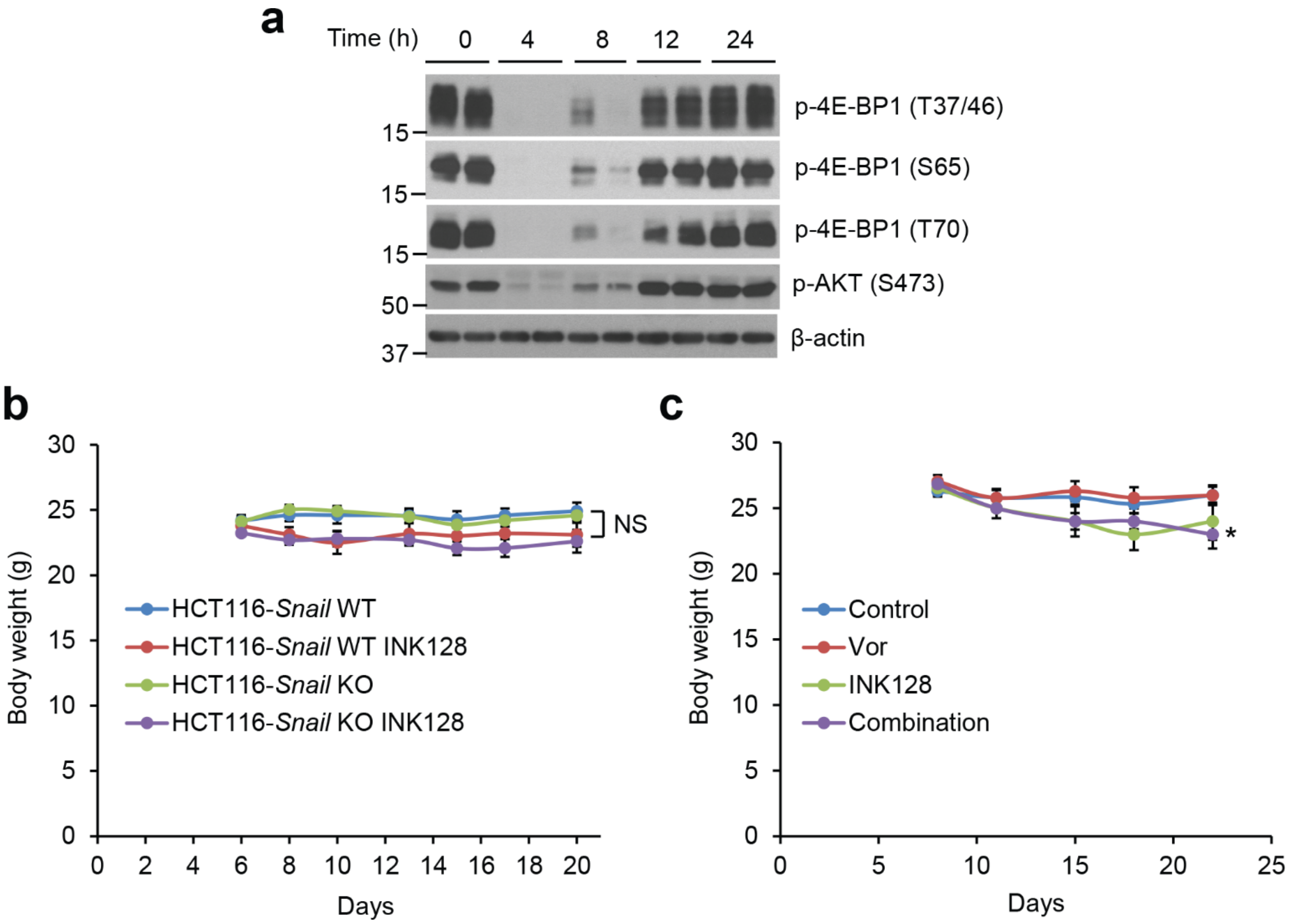

Supplementary Figure 11. Effect of INK128 alone or in combination with vorinostat on 4E-BP1 phosphorylation and weight loss *in vivo*. (a) Pharmacodynamic effect of INK128 on the phosphorylation of 4E-BP1 and AKT *in vivo*. Mice bearing HCT116 xenograft tumors were treated with INK128 at 1.5 mg kg^{-1} for the indicated time periods. Tumors were lysed and analyzed by western blotting for the indicated proteins. (**b**) Mice bearing HCT116-*Snail* WT or knockout (KO) xenograft tumors were treated with INK128 at 1.5 mg kg^{-1} or with vehicle control twice per day for 5 consecutive days each week; mouse body weight was measured twice per week. (**c**) Mice bearing HCT116 xenografts were treated with vorinostat (50 mg kg^{-1}), INK128 (1.5 mg kg^{-1}), the combination of both drugs, or with the vehicle controls once daily for 5 consecutive days each week; mouse body weight was measured twice per week. The results are presented as the mean body weight ± SEM (n=6 mice/group). $^{*}P < 0.04$ for combination vs. control; NS, not significant using Student's *t*-test.

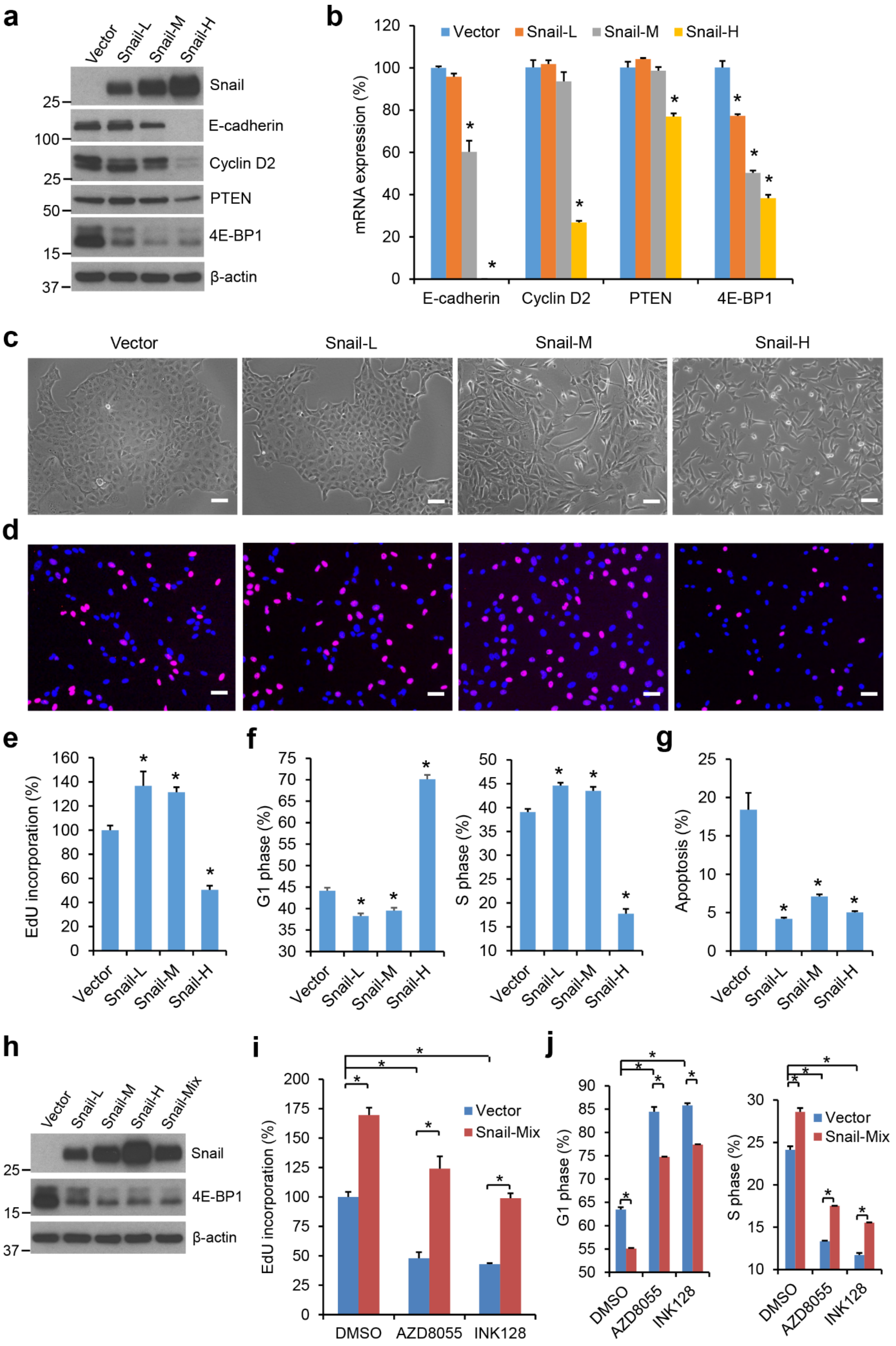
a
Vector
Snail-L
Snail-M
Snail-H
Snail
E-cadherin
Cyclin D2
PTEN
4E-BP1
β-actin
25
100
25
50
15
37
b
Vector
Snail-L
Snail-M
Snail-H
mRNA expression (%)
E-cadherin
Cyclin D2
PTEN
4E-BP1
c
Vector
Snail-L
Snail-M
Snail-H
d
e
EdU incorporation (%)
f
G1 phase (%)
S phase (%)
g
Apoptosis (%)
h
Vector
Snail-L
Snail-M
Snail-H
Snail-Mix
Snail
4E-BP1
β-actin
i
EdU incorporation (%)
Vector
Snail-Mix
DMSO
AZD8055
INK128
j
G1 phase (%)
S phase (%)
Vector
Snail-Mix
DMSO
AZD8055
INK128

Supplementary Figure 12. Effect of different expression levels of Snail on MDCK cell proliferation and survival. (**a, b**) MDCK cells were infected with lentivirus using the pLenti6.3-human Snail or the empty vector construct. Individual cell clones with different expression levels of Snail (L, low; M, medium; H, high) were isolated after selection, and assessed by western blot analysis for the indicated proteins (**a**), or by quantitative RT-PCR analysis for mRNA expression of the indicated genes (**b**). (**c**) Phase-contrast images of living, subconfluent individual MDCK cell clones in culture with low, medium and high Snail expression levels, and their vector control cells. (**d, e**) MDCK cells with low, medium and high Snail expression levels and their vector control cells in culture were treated with EdU for 1 h to identify newly synthesized DNA through fluorescent labelling with a bright, photostable Alexa Fluor® dye in a fast, highly-specific click reaction. DNA was stained with DAPI. Representative images of the indicated cells labelled with EdU (red) and DAPI (blue) are shown in (**d**). The EdU incorporation rate was calculated as a percentage of EdU-positive cells relative to the total number of cells per field, and the results (**e**) are expressed as a percentage of EdU-positive cells relative to values from the vector control cells. (**f**) Flow cytometry analysis of the cell cycle from MDCK cells with low, medium and high Snail expression levels, and their vector control cells, in culture medium with 10% serum. (**g**) MDCK cells with low, medium and high Snail expression levels, and their vector control cells, were serum-depleted for 48 h, followed by flow cytometry analysis using the Annexin V-APC apoptosis detection kit. (**h**) Individual MDCK cell clones with low, medium and high Snail expression levels, or the mixed stable Snail-expressing MDCK cell populations (Snail-Mix), and their vector control cells, were assessed by western blot analysis for the indicated proteins. (**i, j**) The mixed stable Snail-expressing MDCK cells and their vector control cells were treated with 100 nM AZD8055, 100 nM INK128 or DMSO (control) for 24 h, followed by EdU incorporation to assess newly synthesized DNA as described in **d,** and the results are expressed as a percentage of EdU-positive cells relative to values from the DMSO-treated vector control cells (**i**), or by cell cycle analysis using flow cytometry (**j**). Results similar to those presented here (**a-h**) were obtained with other clones of MDCK Snail-L, Snail-M and Snail-H. All graphic data are presented as mean ± SEM (n=3 technical replicates per condition). $^*P < 0.01$ for Snail-L, Snail-M and Snail-H vs. vector or the indicated groups using Student's *t*-test. Scale bar, 100 μm.

Supplementary Figure 13. Uncropped scanned images shown in the indicated figures.

Fig. 1a

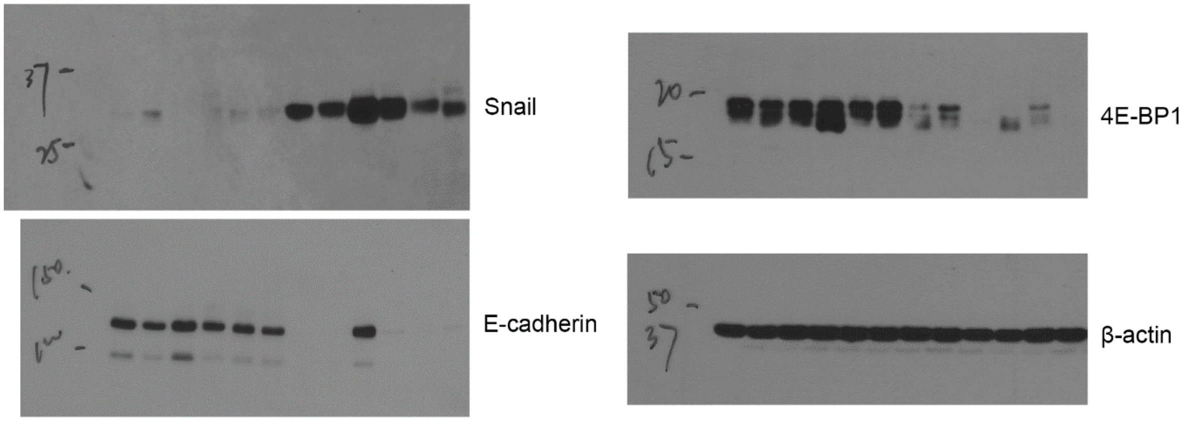

Fig. 2a

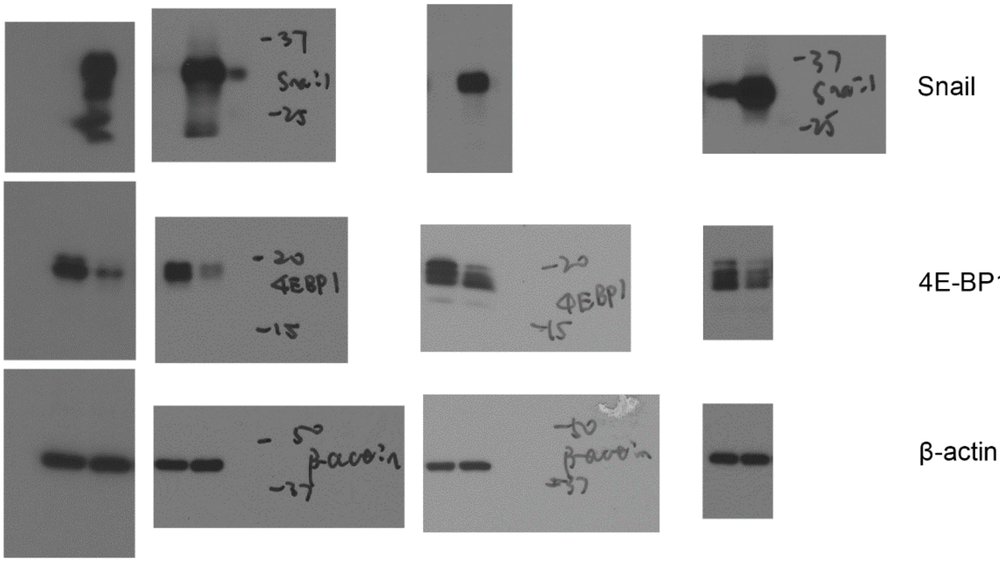

Fig. 2c

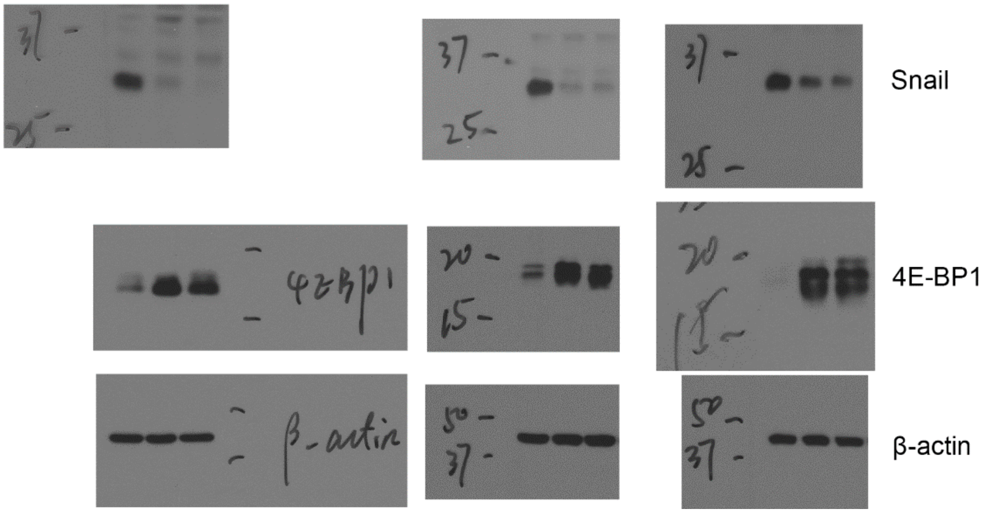

Fig. 3d

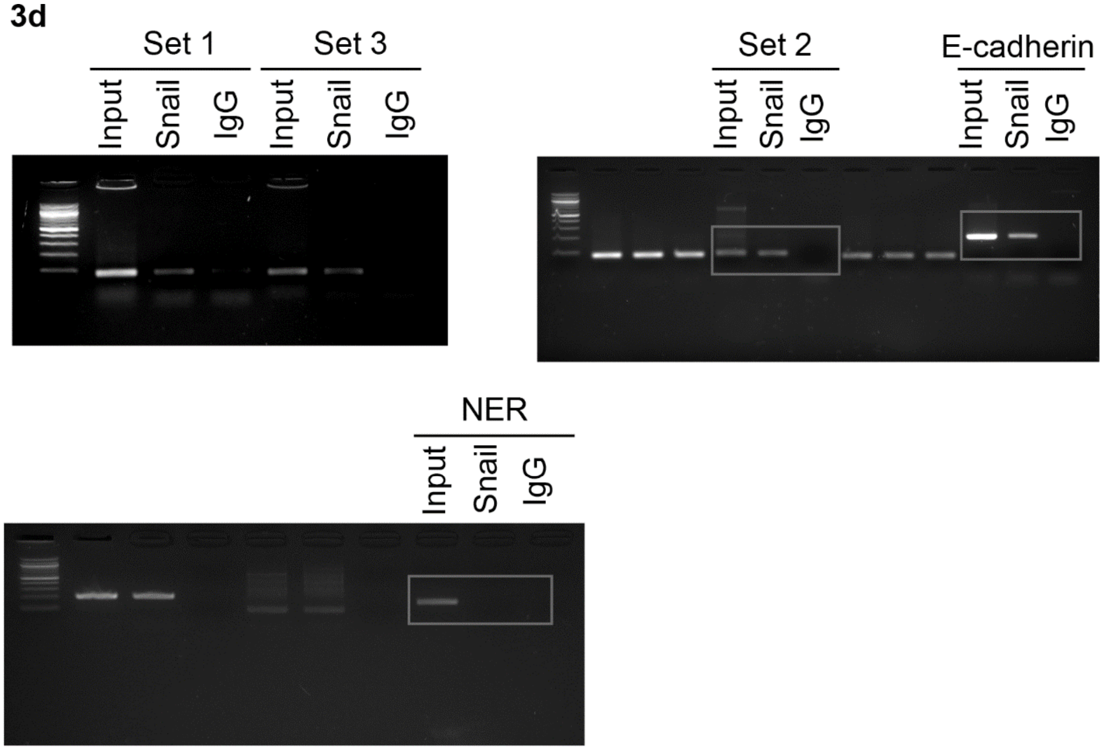

Set 1
Set 3
Input
Snail
IgG
Input
Snail
IgG
Set 2
E-cadherin
Input
Snail
IgG
Input
Snail
IgG
NER
Input
Snail
IgG

Fig. 3f

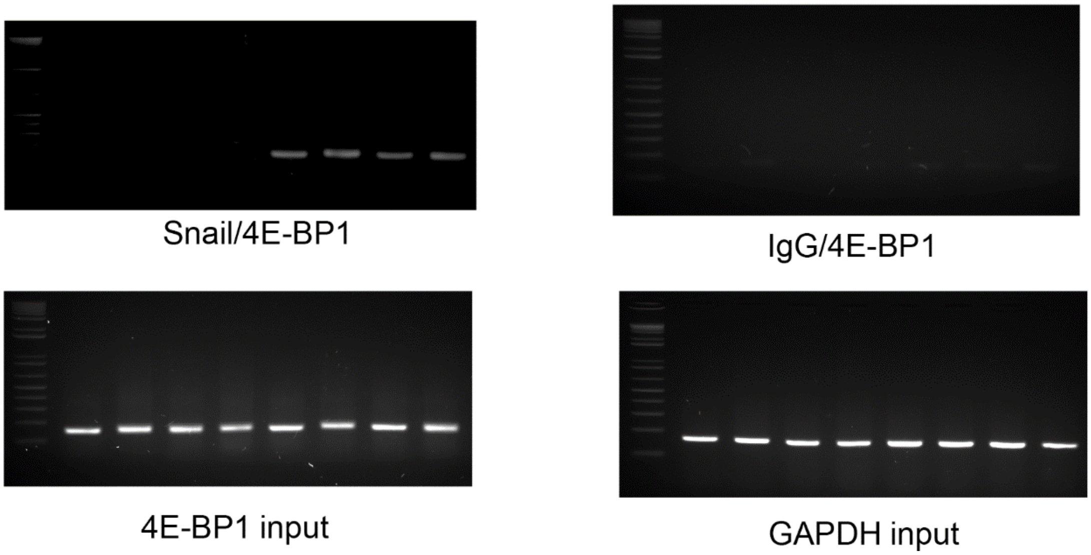

Snail/4E-BP1
IgG/4E-BP1
4E-BP1 input
GAPDH input

Fig. 4d

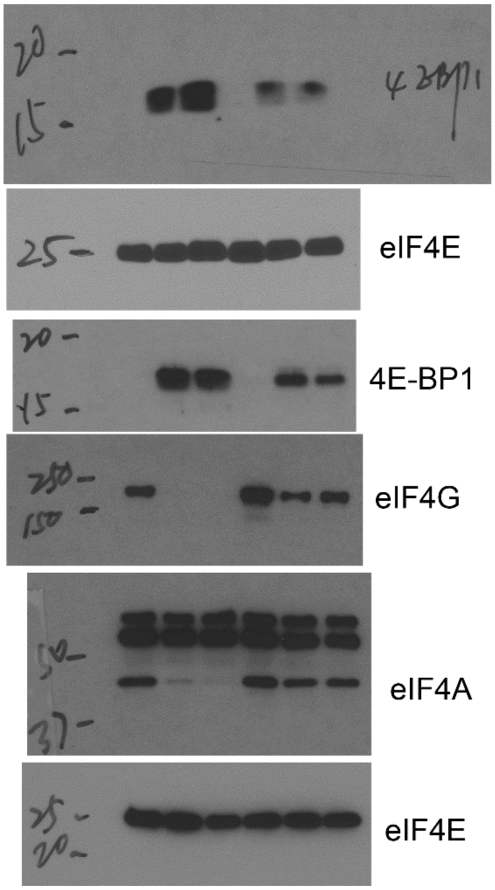

Fig. 4e

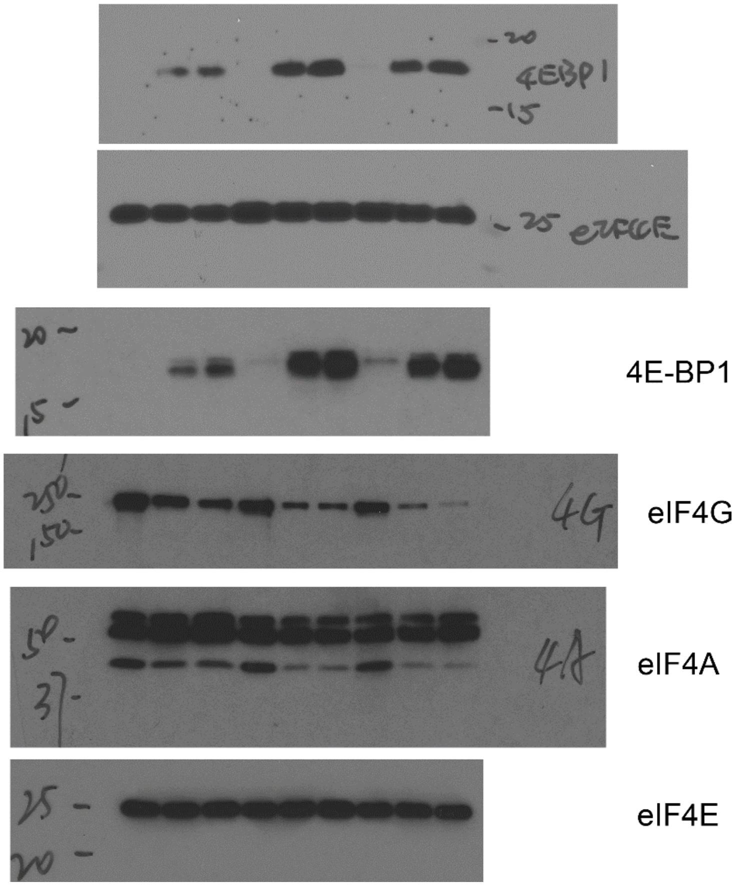

Fig. 6a

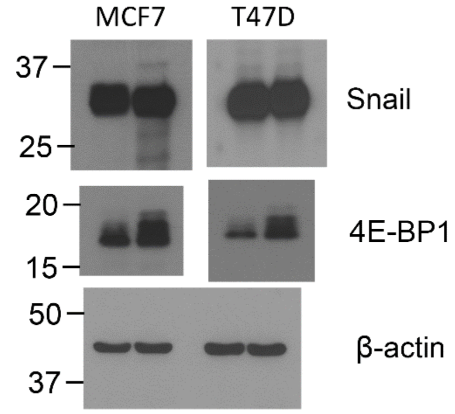

Fig. 6d

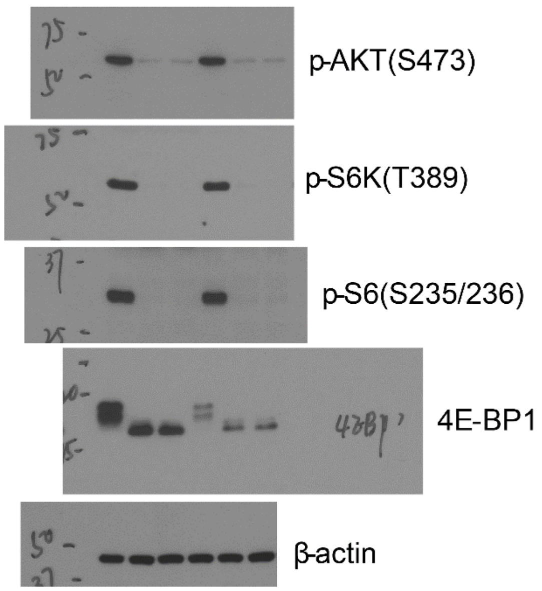

Fig. 6e

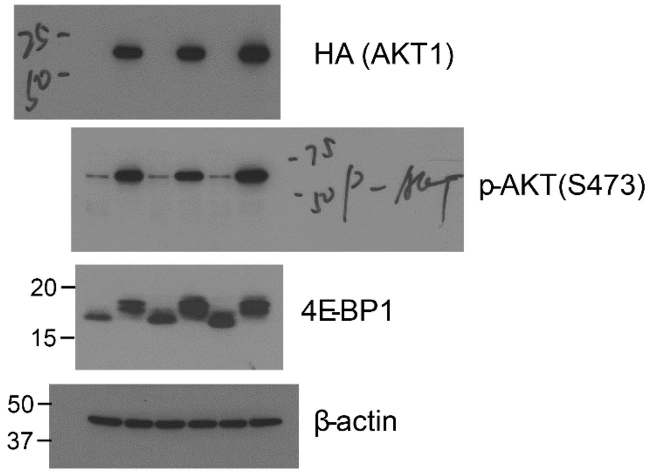

Fig. 6g

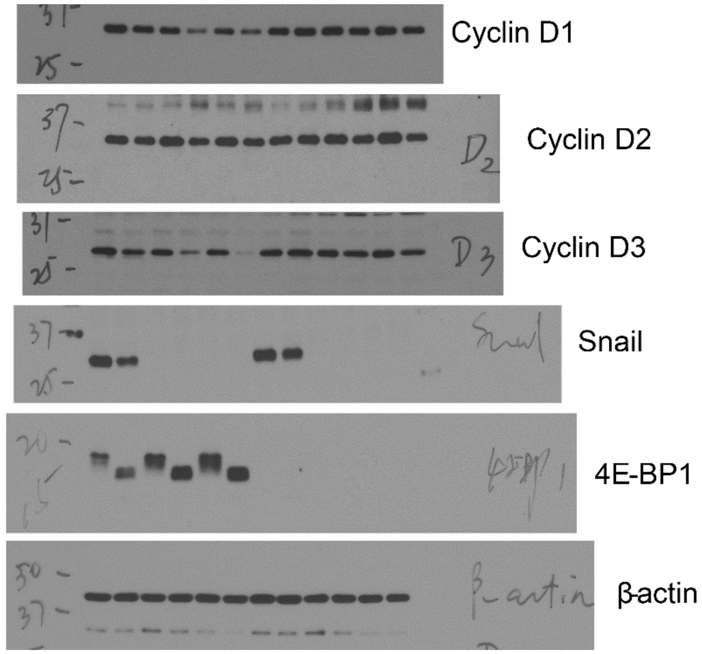

Fig. 7c

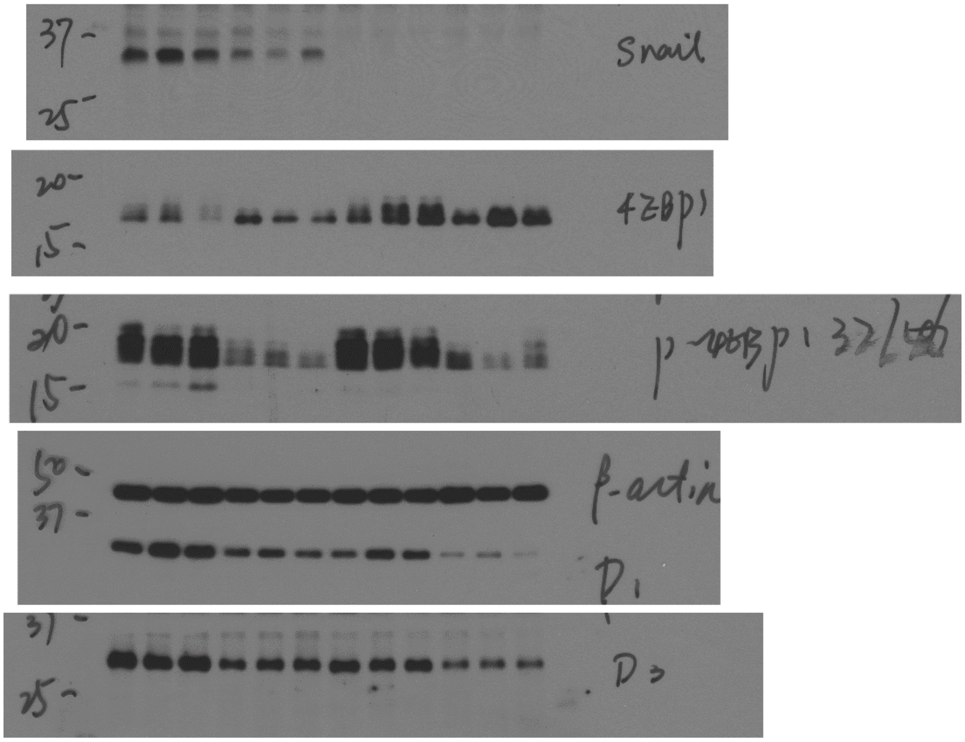

Fig. 7g

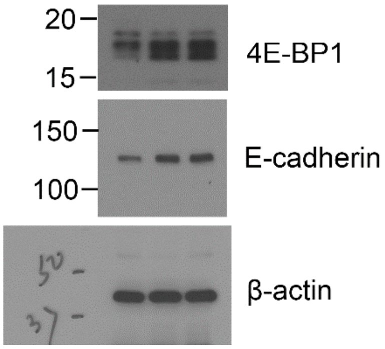

Fig. S1b

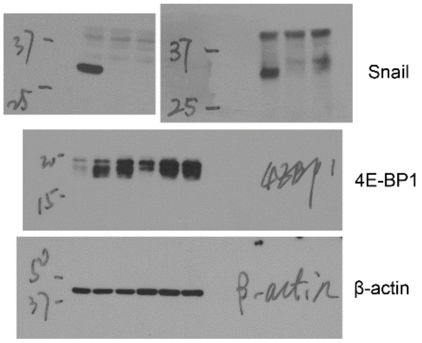

Fig. S1c

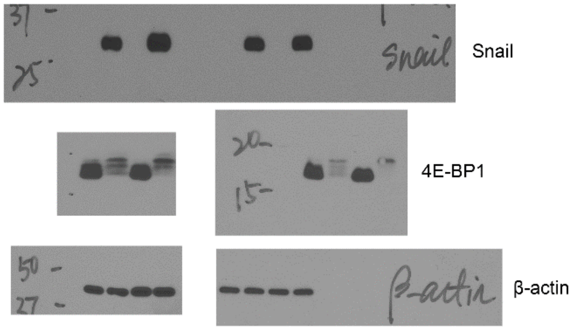

Fig. S2a

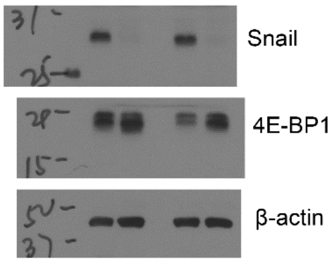

Fig. S4d

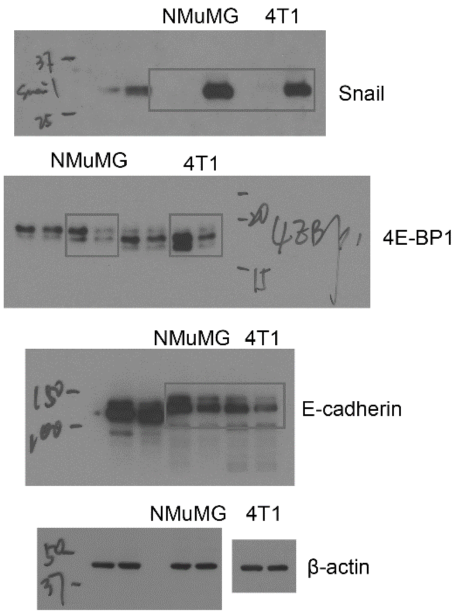

Fig. S4f

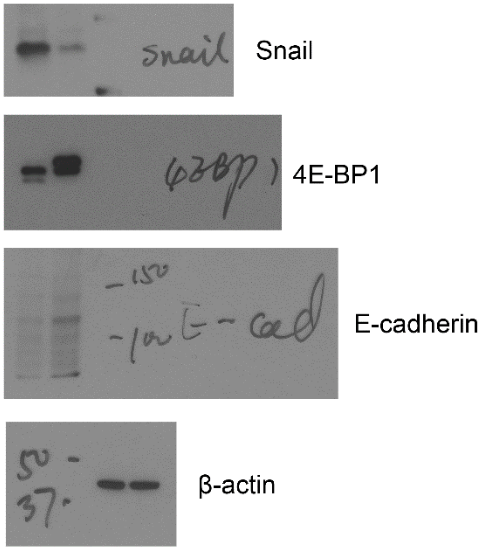

Fig. S5c

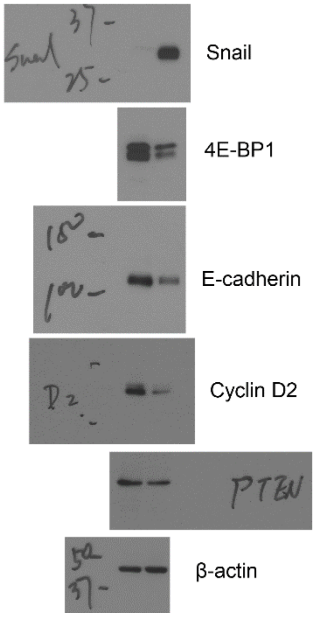

Fig. S6a

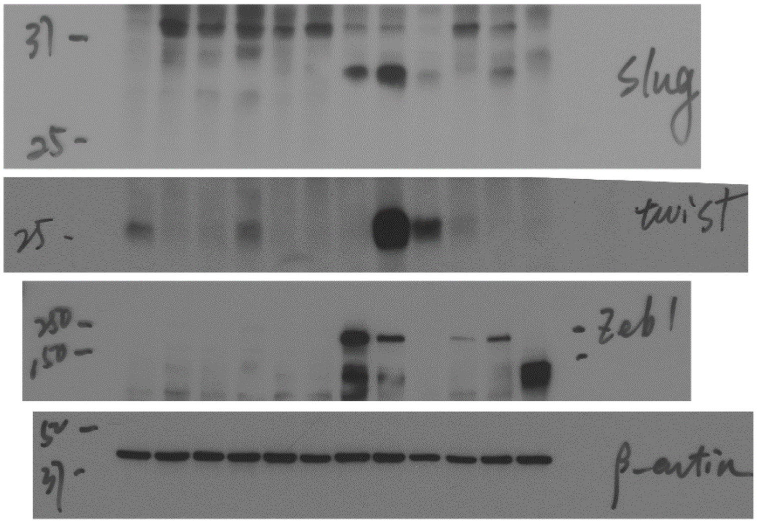

Fig. S6c

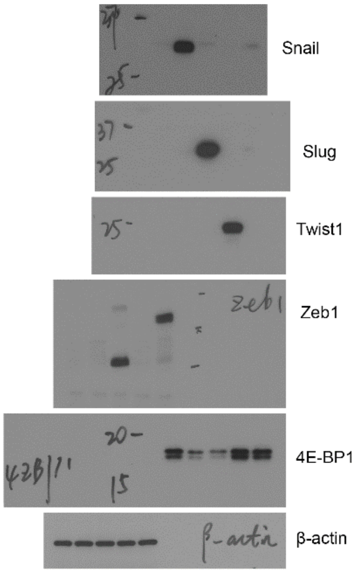

Fig. S6e

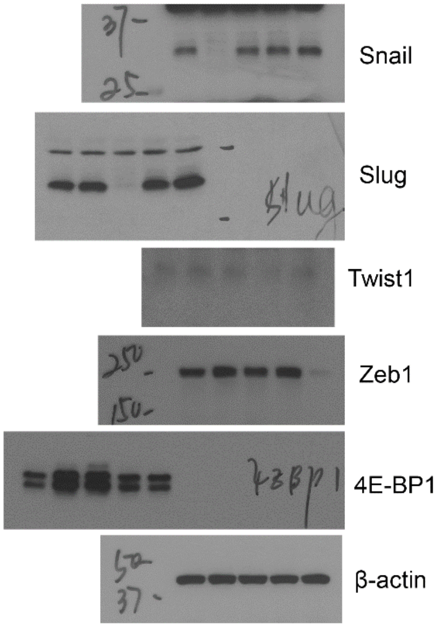

Fig. S6g

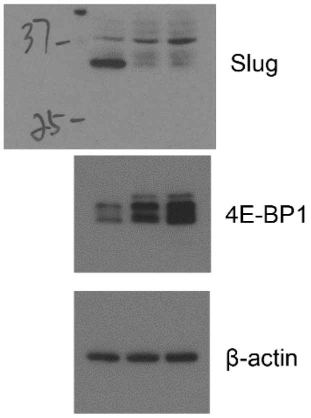

Fig. S6i

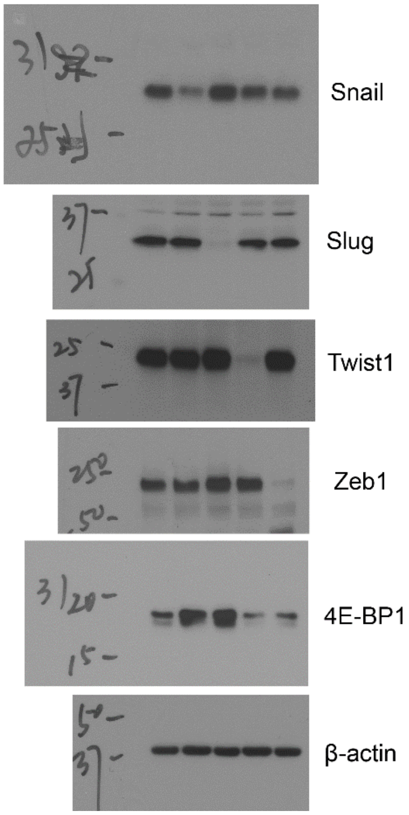

Fig. S7c

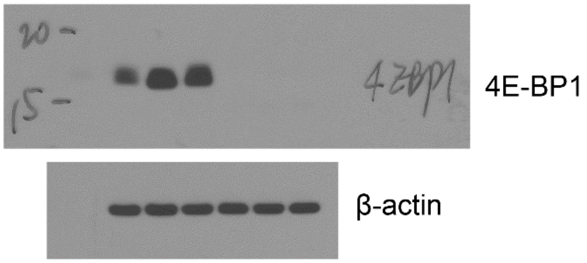

Fig. S9a

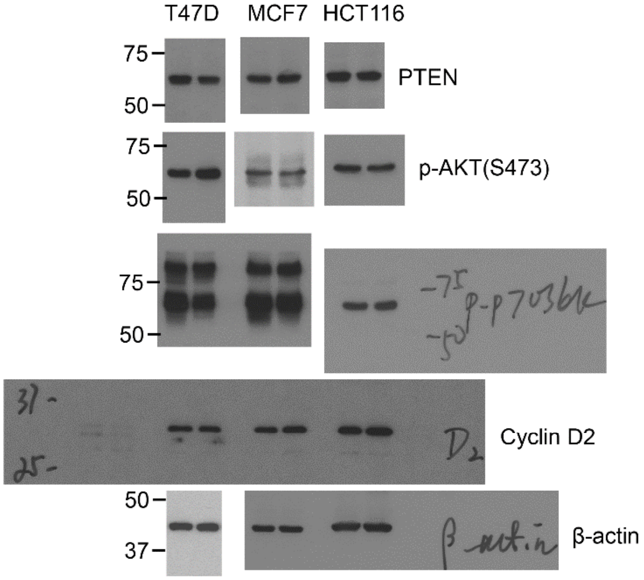

Fig. S9c

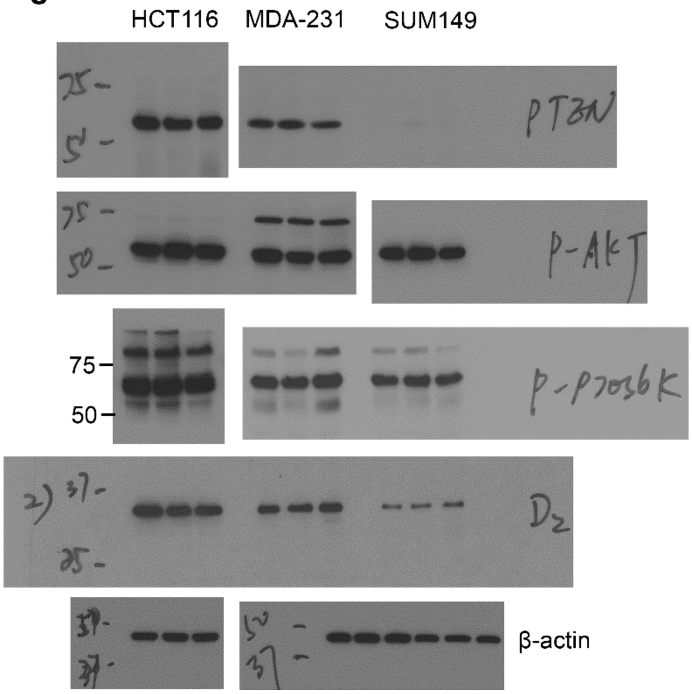

Fig. S10

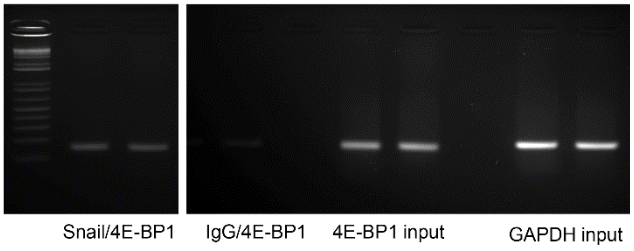

Fig. S11a

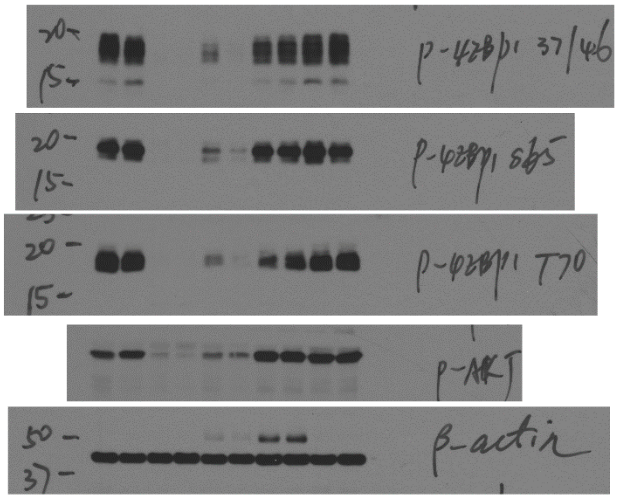

Fig. S12a

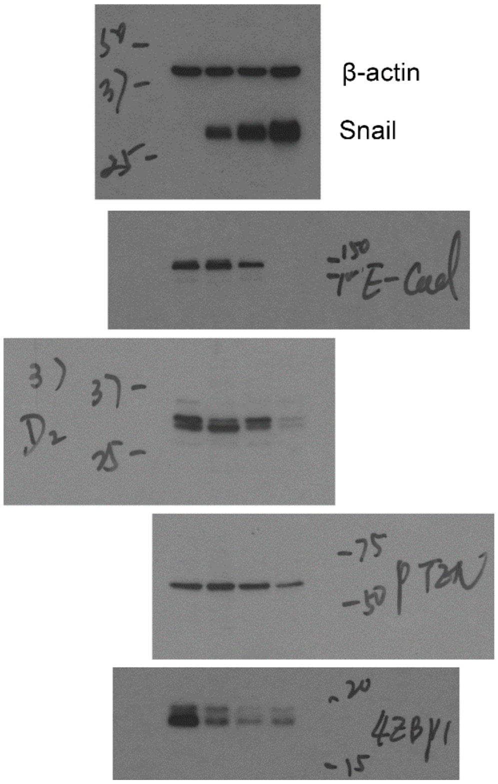

Fig. S12h

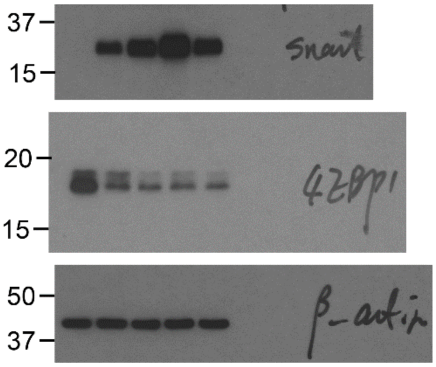

Supplementary Table 1. Primers for 4E-BP1 promoter and its mutant constructs

Species	Constructs	Forward Primers (5'-3')	Reverse Primers (5'-3')
Human	4E-BP1 promoter WT	CAGGTTCAAGCAATTCTCCTG	GGTCTCCTGTGCGCTGCA
	4E-BP1 promoter E1 mut	TGGCTAAGACAGTAGCCACCAGCTACCGTTACACATTGAGCACTCCAAATGTGACC	GGTCACATTTGGAGTGCTCAATGTGTAACGGTAGCTGGTGGCTACTGTCTTAGCCA
	4E-BP1 promoter E2 mut	GGGAAATTGGGTGGTGCCCTTATAATCCCAGCTACTCAGGAAGCTGAGGCTG	CAGCCTCAGCTTCCTGAGTAGCTGGGATTATAAGGGCACCACCCAATTTCCC
	4E-BP1 promoter E3 mut	TAGCCCCTGAATTGAGTCCAACGTCCCCTTAAGGTTTAGTAGGTACTCCTTGGATG	CATCCAAGGAGTACCTACTAAACCTTAAGGGGACGTTGGACTCAATTCAGGGGCTA
Mouse	4E-BP1 promoter WT	CTGTGTAGCTCTGGCAGTCC	CATGTCTCCTGCACGCCG

Supplementary Table 2. shRNA and gRNA sequences for targeting human and mouse *Snail* gene

Species	Constructs	Target sequences (5'-3')
Human	Snail shRNA_1	AGCGAGCTGCAGGACTCTAAT
	Snail shRNA_2	TGAGTAATGGCTGTCACTTGT
	Snail gRNA_1	GGTCGGAGGGCTTCCTGACG
	Snail gRNA_2	GCCTAACTACAGCGAGCTGC
Mouse	Snail shRNA	GCCACCTTCTTTGAGGTACAA

Supplementary Table 3. Primers for real-time quantitative PCR assays

Species	Genes	Forward Primers (5'-3')	Reverse Primers (5'-3')
Human	4E-BP1	GGAACTCACCTGTGACCAAAA	CCGCTTATCTTCTGGGCTATT
	4E-BP2	GTTGGATCGTCGCAATTCTC	TGCATGTTTCCTGTCGTGAT
	4E-BP3	TACGACCGAAAGTTCCTGCT	GAGAGAGGGGCTGTTGGAG
	E-cadherin	CAGCACGTACACAGCCCTAA	GGCGTTGTCATTCACATCAG
	Cyclin D1	TCCTCTCCAAAATGCCAGAG	GGCGGATTGGAAATGAACTT
	Cyclin D3	CTGTGCATCTACACCGACCA	GAATGAAGGCCAGGAAATCA
	GAPDH	ACAACTTTGGTATCGTGGAAGG	GCCATCACGCCACAGTTTC
Mouse	4E-BP1	GGTCACTAGCCCTACCAGCG	TTGTGACTCTTCACCGCCTG
	E-cadherin	GGGGAAGCGGTGGAGGATC	AGGCGATGGCAGCGTTGTAG
Canine	4E-BP1	GTCGGAACTCACCTGTGACC	ACTGTGACTCTTCACCACCG
	E-cadherin	CTAATGGGAATGCGGTTGAAG	GGCAGCGTTGTAGGTATTCAC
	Cyclin D2	TCATCGAGCACATCCTTCGG	GCGAACTTAAAGTCGGTGGC
	PTEN	GGTTTCCTGCAGAAAGACTTG	TAGCTGTGGTGGATTATGGTC
Mouse and Canine	GAPDH	GTCCATGCCATCACTGCCAC	TGACCTTGCCCACAGCCTTG

Supplementary Table 4. Primers for ChIP assay

Species	Genes	Forward Primers (5'-3')	Reverse Primers (5'-3')
Human	4E-BP1 (set 1)	AACTTGTGACAGTTTGGACTGG	GGCCTGCAGTGGCTAAGACAG
	4E-BP1 (set 2)	GCCTCAGCTTCCTGAGTAGC	TATTGTGGGGAAGAGGGTGA
	4E-BP1 (set 3)	CATCCATCCATCCAAGGAGT	GAAGAGGTTTCCCGATAGCC
	4E-BP1 (NER)	TCCTCCCACACCCTCTCTAA	TCCCTGTTTCGTGCCTTAGT
	E-cadherin	TAGAGGGTCACCGCGTCTAT	TCACAGGTGCTTTGCAGTTC
